# Adjunctive granisetron therapy in patients with sepsis or septic shock (GRANTISS): A single-center, single-blinded, randomized, controlled clinical trial

**DOI:** 10.3389/fphar.2022.1013284

**Published:** 2022-12-13

**Authors:** Jianbin Guan, Yuping Liao, Yuexun Guo, Shuang Yu, Rongjuan Wei, Mengwei Niu, Jianwei Gan, Lu Zhang, Tong Li, Jin Lv, Maoyou Shichen, Ping Chang, Peng Chen, Zhanguo Liu

**Affiliations:** ^1^ Department of Critical Care Medicine, Zhujiang Hospital, Southern Medical University, Guangzhou, China; ^2^ Department of Critical Care Medicine, Dongguan People’s Hospital, Dongguan, China; ^3^ Department of Critical Care Medicine, DongGuan Tungwah Hospital, DongGuan, China; ^4^ Guangdong Provincial Key Laboratory of Proteomics, State Key Laboratory of Organ Failure Research, Department of Pathophysiology, School of Basic Medical Sciences, Southern Medical University, Guangzhou, China

**Keywords:** sepsis, granisetron, adjunctive therapy, intensive care, 5-HT3 receptor antagonists

## Abstract

**Background:** In preclinical experiments, we demonstrated that the 5-HT3 receptor antagonist granisetron results in reduced inflammation and improved survival in septic mice. This randomized controlled trial was designed to assess the efficacy and safety of granisetron in patients with sepsis.

**Methods:** Adult patients with sepsis and procalcitonin ≥ 2 ng/ml were randomized in a 1:1 ratio to receive intravenous granisetron (3 mg every 8 h) or normal saline at the same volume and frequency for 4 days or until intensive care unit discharge. The primary outcome was 28-day all-cause mortality. Secondary outcomes included the duration of supportive therapies for organ function, changes in sequential organ failure assessment scores over 96 h, procalcitonin reduction rate over 96 h, the incidence of new organ dysfunction, and changes in laboratory variable over 96 h. Adverse events were monitored as the safety outcome.

**Results:** The modified intention-to-treat analysis included 150 septic patients. The 28-day all-cause mortalities in the granisetron and placebo groups were 34.7% and 35.6%, respectively (odds ratio, 0.96; 95% CI, 0.49–1.89). No differences were observed in secondary outcomes. In the subgroup analysis of patients without abdominal or digestive tract infections, the 28-day mortality in the granisetron group was 10.9% lower than mortality in the placebo group. Adverse events were not statistically different between the groups.

**Conclusion:** Granisetron did not improve 28-day mortality in patients with sepsis. However, a further clinical trial targeted to septic patients without abdominal/digestive tract infections perhaps is worthy of consideration.

## Introduction

Sepsis is characterized by organ dysfunction due to a dysregulated host response to infection (Singer et al., 2016). Despite considerable progress in sepsis treatment over the past decades, the World Health Organization still considers sepsis a primary health threat (Reinhart et al., 2017). Besides high mortality, sepsis incurs high medical care costs. According to a cross-sectional survey, the median hospitalization cost for patients with sepsis in China is as high as ¥91,556 (Xie et al., 2020). In the United States, the average treatment costs for patients with sepsis or septic shock are $24,638 and $38,298, respectively (Paoli et al., 2018). Hence, sepsis imposes a heavy economic burden on the national healthcare system, patients, and their families. Even for those patients who survive and are discharged from the hospital, the complications caused by sepsis affect the long-term quality of daily life (Contrin et al., 2013; Cuthbertson et al., 2013). There is evidence that exploring new cost-effective approaches to sepsis is not only help improve prognosis but also save medical costs (Kyeremanteng et al., 2018). Thus, cheap, safe, effective, and easily available treatments in sepsis are urgently needed.

The extensive and intense inflammatory cytokines are key factors contributing to tissue damage and organ dysfunction during the development of sepsis. Serotonin (5-HT), a neurotransmitter and hormone, participates in the regulation of physiological functions (Berger et al., 2009). Additionally, 5-HT play a crucial role in the pathogenesis of sepsis. During the development of sepsis, the release of a large quantity of 5-HT promotes oxidative stress, nitrifying stress, lipid peroxidation, increased bacterial load, and the production of pro-inflammatory cytokines, which exacerbate organ dysfunction and death in sepsis (Nocito et al., 2007; Ahern, 2011; Duerschmied et al., 2013; Shajib & Khan, 2015; Zhang et al., 2017). The actions of 5-HT are mediated through 5-HT receptors. Hence, 5-HT receptor antagonists may block the abnormal inflammatory response in sepsis. Tropisetron, a 5-HT3 receptor antagonist, inhibited excessive inflammatory cytokine production during the development of severe sepsis/septic shock in a rat sepsis model (Setoguchi et al., 2011). A 5-HT2A receptor antagonist attenuated the inflammatory response, maintained hemodynamic stability, inhibited lung injury, and reduced mortality in an animal model of septic shock (Nishiyama, 2009). In our previous studies, we demonstrated that the 5-HT3 receptor antagonist, granisetron, is a metabolite generated by gut microbiota and released into the circulatory system, resulting in reduced inflammatory necrosis and thrombosis in liver tissue, reduced alveolar pathological damage, and improved survival in a septic mouse model by suppressing the 5-HT3 receptor-AKT/NF-κB, 5-HT3 receptor-p38 and 5-HT3 receptor-NLRP3d-Cxcl1/Cxcl2 signaling pathways (Gong et al., 2019; Wang et al., 2019). More importantly, granisetron was detected in the feces of septic patients, and lower fecal granisetron levels correlated with higher plasma liver injury markers (Gong et al., 2019). Based on this evidence, a 5-HT3 receptor antagonist such as granisetron may reduce organ dysfunction and improve prognosis in patients with sepsis.

Granisetron, a highly selective 5-HT3 receptor antagonist, has been safely used for several years to treat or prevent nausea and vomiting induced by chemotherapy (Spartinou et al., 2017). Based on the efficacy of granisetron for the treatment of sepsis or septic shock in preclinical experiments, we hypothesized that granisetron will improve the outcomes of patients with sepsis. Thus, to initially investigate the safety and effectiveness of granisetron in patients with sepsis, we performed a single-center, single-blinded, randomized, placebo-controlled trial (GRANTISS).

## Methods

### Study design and study setting

GRANTISS is a single-center, single-blinded, randomized, placebo-controlled trial conducted by the Department of Critical Care Medicine of Zhujiang Hospital in Guangzhou, China. The trial followed the 1964 Declaration of Helsinki and the subsequent amendments. The study protocol was approved by the Clinical Trial Committee of Zhujiang Hospital and the Clinical Ethics Committee of Zhujiang Hospital of Southern Medical University (2018-ZZJHZX-009). Information on the trial was registered on ClinicalTrials.gov (NCT03924518), and a detailed protocol was previously published (Guan et al., 2019).

### Population

Adult patients, eighteen to 80 years old, meeting the sepsis 3.0 diagnostic criteria with procalcitonin (PCT) ≥ 2 ng/ml were eligible for inclusion in the study (Singer et al., 2016; Marik et al., 2017). PCT of 2 ng/ml was used as a threshold because it was thought to increase the certainty of sepsis and sepsis-related organ dysfunction (Marik et al., 2017; Pontrelli et al., 2017). There is a need to eliminate the possible death bias mainly due to combined diseases such as terminal illnesses, irreversible or severe immunodeficiencies, which are more likely to result in death than sepsis. We excluded the patients if they had any of the following conditions: a solid organ or bone marrow transplant; myocardial infarction within the past 3 months; advanced pulmonary fibrosis; cardiopulmonary resuscitation before enrollment; HIV-positive; neutropenia; non-remission blood/lymphatic system tumors; limited care; long-term history of immunosuppressive drugs; immunodeficiency; advanced tumors; non-infectious factors leading to death (uncontrolled large bleeding, cerebral herniation, *etc.*); unresolved infection sources from puncture, drainage, debridement, or other surgical procedures; allergy to granisetron; or intestinal obstruction (Guan et al., 2019). In addition, we also excluded pregnant or lactating women due to safety consideration. All patients included in the study provided written informed consent.

### Randomization and blinding

Study participants were randomly assigned to the granisetron group or the control group in a 1:1 ratio. A random sequence with a block size of eight was generated by an independent Data Security Management Board using Stata 13. According to the random sequence, group cards marked with group information (granisetron group or placebo group) were produced for each patient. Each card was put into a sealed envelope. An envelope was opened for each patient at the time of enrollment to complete the random assignment. When multiple patients were successfully recruited at the same time, the order of enrollment was determined according to the time of intensive care unit (ICU) admission. Normal saline with the same appearance as granisetron was used as the placebo. Granisetron or placebo was continuously infused at the same rate with identical 50 ml syringes. During the trial, the patients and their relatives were blinded but the treating clinicians were not blinded to the trial regimen.

### Intervention

Patients randomly assigned to the granisetron group received 3 mg of intravenous granisetron every 8 h for 4 days or until ICU discharge. Patients randomly assigned to the control group received normal saline at the same volume, frequency, and duration. All patients received routine treatment according to the 2016 International Management Guidelines for Sepsis, including but not limited to infection control as soon as possible after diagnosis, early fluid resuscitation and fluid management guided by hemodynamic monitoring, vasopressors to maintain the mean arterial pressure when fluid therapy failed, early recovery of enteral nutrition, standardized supportive therapies for organ function such as invasive mechanical ventilation (MV) and continuous renal replacement therapy (CRRT), and prevention of complications.

### Study outcomes

The primary outcome was all-cause mortality on the 28th day. The secondary outcomes included the duration of supportive therapies for organ function, including MV, CRRT, and vasopressor; the length of ICU stay (LOS); mean changes in sequential organ failure assessment scores (SOFA) over 96 h; the reduction rate of PCT over 96 h, which was defined as the difference between initial PCT minus PCT at 96 h divided by the initial PCT multiplied by 100 (Marik et al., 2017); the incidence of new organ dysfunction; and mean changes in laboratory variables over 96 h, including alanine transaminase (ALT), aspartate aminotransferase (AST), total bilirubin, interleukin-6, C-reactive protein, erythrocyte sedimentation rate, white blood cell count, lymphocyte count, Cystatin C, serum creatinine, blood urea nitrogen, blood lactate, superoxide dismutase, and oxidation index (PaO2/FIO2). Safety was evaluated up to 28 days after enrollment, including analysis of all adverse events and serious adverse events.

### Statistical analyses

The mortality due to sepsis in the study center is approximately 33% (Chang et al., 2020). As an initially exploratory trial, the sample size was estimated to allow for the detection of a 20% difference in the 28-day mortality between the groups with 80% power and a two-sided significance level of 5%. The estimated sample size was increased by 15% to compensate for the loss of patients during follow-up. Therefore, 154 patients with sepsis (77 per group) were needed to show statistical differences.

Patients who received at least one dose of granisetron or placebo and one outcome assessment were included in the modified intention-to-treat analysis for primary and secondary outcomes. All patients were included in the safety analysis. The descriptive continuous variables are presented as means and standard deviations or medians and interquartiles. The categorical variables are presented as frequencies and composition ratios. The primary outcome was analyzed using logistic regression. The survival information of patients is presented as a Kaplan–Meier plot, and the Cox model was used to compare the effects of study agents. Primary outcomes were also compared in four subgroups analyses determined by the following criteria: Acute Physiology and Chronic Health Evaluation II score (APACHE II) ≤ 25 vs. >25, age ≤65 vs. >65 years, the time of sepsis diagnosis at recruitment ≤48 h vs. >48 h, and digestive/abdominal infection vs. no digestive/abdominal infection. Digestive/abdominal tract infection was defined as the presence of evidence from microbial culture, imaging, or clinical signs. For secondary outcome comparisons, continuous variables with a normal distribution were analyzed with a two-sample *t* test or t’ test. Continuous variables with a non-normal distribution were compared using a Wilcoxon rank-sum test. Mean differences in repeated measure variables between groups were analyzed using a mixed linear model that included the time points (baseline, 24, 48, 72, and 96 h), groups (granisetron or placebo), and the interaction between groups and time points. The Pearson chi-square test was used to compare qualitative variables and adverse events. A threshold of *p* < 0.05 (two-sided) was considered statistically significant.

## Results

**FIGURE 1:**
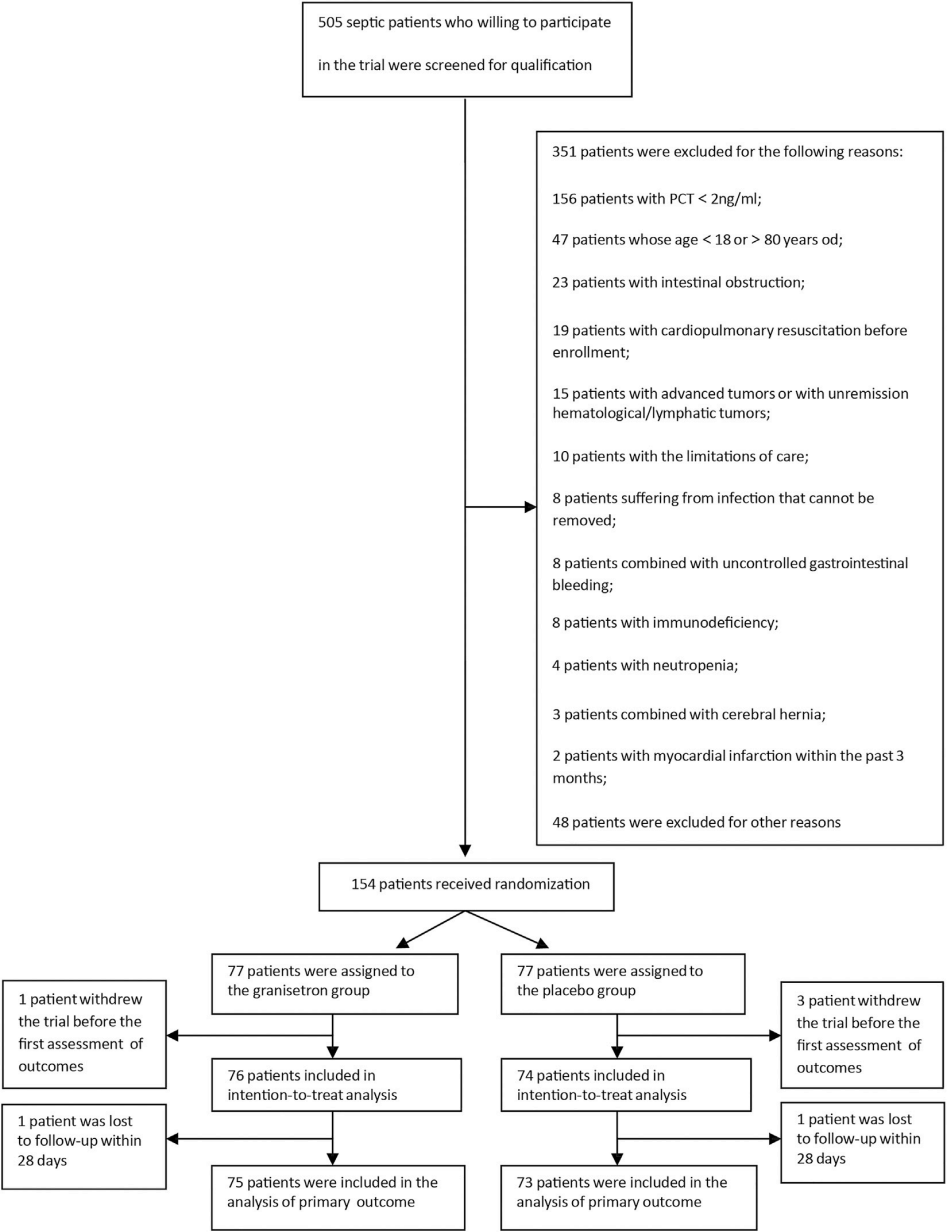
Flow chart of patient enrollment.

From April 2019 to November 2020, we screened 505 patients admitted to the ICU with sepsis. 351 of these septic patients met the exclusion criteria. Hence, we successfully enrolled 154 patients. The modified intention-to-treat analysis included 76 patients in the granisetron group and 74 patients in the placebo group. Except for two patients who were lost to follow-up within 28 days, 148 patients were included in the primary outcome analysis (Figure 1). All comparisons between groups used the placebo as a reference.

Table 1 shows the baseline characteristics of patients, which were similar between groups. Most patients had comorbidities at ICU admission, including hypertension (granisetron vs. placebo: 39.5% vs. 37.8%), diabetes (26.3% vs. 28.4%), acute kidney failure (30.3% vs. 28.4%), and liver diseases (34.2% vs. 25.6%). Most patients needed supportive therapies for organ function. More than two-thirds of the patients received MV and more than one-third of the patients received CRRT. Approximately 50% of patients received vasopressors. No significant differences in the laboratory variables were detected. The SOFA scores (9.3 ± 4.2 vs. 9.7 ± 4.4, *p* = 0.63) and APACHE II scores (20.7 ± 7.8 vs. 22.4 ± 7.8, *p* = 0.20) were similar between the two groups.

**TABLE 1:** Clinical characteristics of enrolled patients with sepsis.

Variable	Granisetron (n = 76)	Placebo (n = 74)	*p*-Value
Age, median (IQR), year	60 (47.8, 65.3)	58 (48.3, 67.0)	0.80
Sex, male, N (%)	58 (76.3)	56 (75.7)	1
Infection site, N (%)			
Pulmonary infection	53 (69.7)	51 (68.9)	0.91
Digestive and abdominal infection	15 (19.7)	19 (25.6)	0.39
Urinary infection	2 (2.6)	4 (5.4)	0.39
Skin and soft tissue infection	6 (7.9)	7 (9.5)	0.73
Intracranial infection	1 (1.3)	2 (2.7)	0.54
Bloodstream infection	8 (10.5)	10 (13.5)	0.57
Unknown site	7 (9.2)	3 (4.1)	0.21
Comorbidities, N (%)			
Hypertension	30 (39.5)	28 (37.8)	0.83
Diabetes	20 (26.3)	21 (28.4)	0.78
CHD or HF	21 (27.6)	11 (14.9)	0.06
Cerebrovascular accident	8 (10.5)	11 (14.9)	0.42
Acute kidney failure	23 (30.3)	21 (28.4)	0.80
Chronic renal failure	12 (15.8)	8 (10.8)	0.36
Liver disease	26 (34.2)	19 (25.6)	0.25
Other	7 (9.2)	5 (6.8)	0.57
Organ function support			
Mechanical ventilation, N (%)	55 (72.4)	62 (83.8)	0.14
Vasopressors, N (%)	35 (46.1)	39 (52.7)	0.51
CRRT, N (%)	25 (32.9)	28 (37.8)	0.64
ECMO, N (%)	1 (1.3)	1 (1.4)	0.98
concomitant with other drugs			
Ulinastatin, N (%)	9 (11.8)	6 (8.1)	0.45
Methylprednisolone, N (%)	14 (18.4)	12 (16.2)	0.72
Hydrocortisone	9 (11.8)	6 (8.1)	0.45
Laboratory examination			
WBC, median (IQR), ×10^9^	13.3 (9.3,17.8)	14.3 (9.1,20.1)	0.60
Lymphocyte count, Median (IQR), ×10^9^	0.90 (0.59,1.40)	0.88 (0.57,1.30)	0.89
PCT, median (IQR), ug/L	9.5 (5.2,37.2)	21.1 (6.4,55.6)	0.13
Interleukin-6, median (IQR), ng/L	110.5 (51.0,465.8)	147.0 (54.6,447.2)	0.90
ESR, median (IQR), mm/h	40.5 (15.3,72.0)	46 (26.5,83.5)	0.18
CRP, median (IQR), ng/L	120.0 (73.4,186.1)	148.5 (93.1,212.8)	0.06
Blood lactate, median (IQR), mmol/L	1.8 (1.2,2.8)	1.9 (1.3,3.0)	0.61
Cys C, median (IQR), mg/L	1.46 (1.1,2.1)	1.56 (1.1,2.3)	0.63
SCr, median (IQR), umol/L	162.0 (76.3,290.4)	140.0 (92.5,271.0)	0.89
TBIL, median (IQR), umol/L	20.2 (11.3,66.9)	19.5 (10.8,44.7)	0.29
ALT, median (IQR), IU/L	44.0 (23.0,102.0)	52.5 (19.3,139.5)	0.62
AST, median (IQR), IU/L	77.0 (32.0,152.5)	76.0 (30.8,221.0)	0.65
5-HT^a^, median (IQR), ng/L^a^	38.6 (17.5,76.8)	44.4 (20.8,87.4)	0.43
PaO2/FiO2, median (IQR)	194.6 (161.2,290.0)	217.1 (160.5,297.1)	0.267
SOFA score, mean (SD)	9.3 (4.2)	9.7 (4.4)	0.63
APACHE II score, mean (SD)	20.7 (7.8)	22.4 (7.8)	0.20

Data are presented as values (%), mean (SD), or median (interquartile range). a: nine samples were not tested due to damage, and a total of 141 serum samples were tested for serum 5-HT, baseline concentration (71 in the treatment group and 70 in the control group).APACHE II, acute physiology and chronic health evaluation; ALT, alanine transaminase; AST, aspartate aminotransferase; CHD, coronary heart disease; CRP, C-reactive protein; Cys C, cystine C; CRRT, continuous renal replaced treatment; ESR, erythrocyte sedimentation rate; ECMO, extracorporeal membrane oxygenation; HF, heart failure; SCr, serum creatinine; SOFA, sequential organ failure assessment; TBIL, total bilirubin; PCT, procalcitonin; WBC, white blood cell; 5-HT, 5-hydroxytryptamine.

**FIGURE 2:**
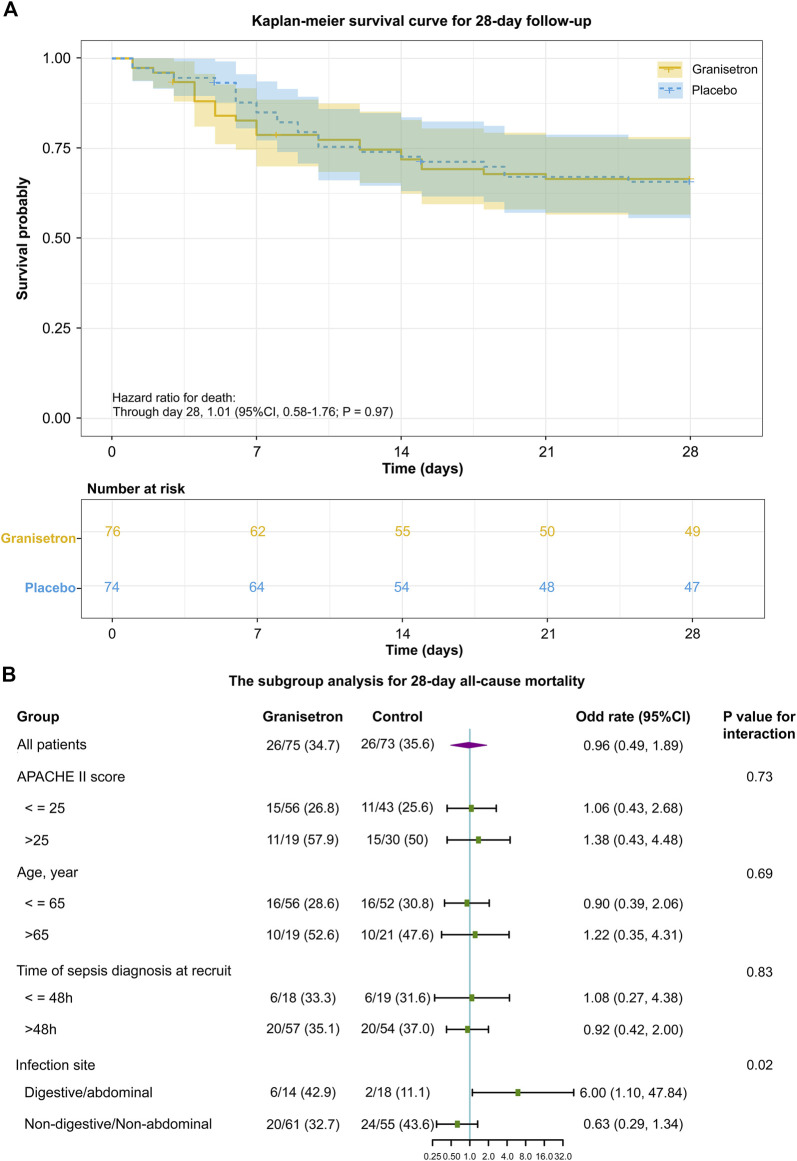
The primary outcome of granisetron therapy in all patients or in patients of the different subgroup **(A)** Kaplan–Meier survival curve during the 28 day follow-up. In the survival analysis, 150 patients were included. The date of mortality for two patients within 28 days was not available, so it was censored. The tick marks indicate censored data. The *p*-value was calculated using a Cox proportional hazards model that included the randomized trial group. **(B)** The subgroup analysis for 28 day all-cause mortality. The variables of the subgroup analysis, odd rate, 95% CI in each comparison, the number of patients (denominator), the number of deaths (numerator) in each subgroup, and the *p*-value of interaction between the groups (granisetron or placebo) and the grouped variables (APACHE II score or Age or Time of sepsis diagnosis at recruit or Infection site) are shown.

All patients were enrolled within 24 h of ICU admission, except for patients with new-onset sepsis during ICU admission. The median time from ICU admission to the first trial regimen was 19 h (16.5–23.0) *versus* 17.5 h (15.5–21.0), and the average number of doses per patient was 10.4 ± 2.9 and 10.6 ± 2.7 for granisetron group and placebo group, respectively. Twenty-six patients in each group died during the 28-day trial period; the 28-day mortalities were not significantly different (34.7% vs. 35.6%; Odds Ratio [OR] 0.96; 95% CI 0.49–1.89; *p* = 0.90). The 28-day or 60-day survivals were also similar between the groups (28-day hazard ratio 1.01; 95% CI 0.58–1.76; *p* = 0.97 [Figure 2A]; 60-day hazard ratio 0.86; 95% CI 0.51–1.44; *p* = 0.57) (Supplementary Figure S1). The mean SOFA score improved from 9.3 ± 4.2 to 7.4 ± 4.1 in the granisetron group and from 9.7 ± 4.4 to 8.3 ± 4.8 in the placebo group over 96 h (Mean difference [MD] 0.19, 95% CI −0.60–0.97, *p* = 0.67) (Supplementary Figure S2). Granisetron did not reduce the incidence of new-onset organ dysfunction (17.1% vs. 14.9%; *p* = 0.88) or PCT reduction rate (78.6% vs. 72.7%; *p* = 0.18) over 96 h. No differences in the median duration of vasopressor, MV, and CRRT were detected (Table 2). In contrast to previous experiments, improvements in liver biomarkers and other laboratory variables were not detected in the granisetron group in comparison with the placebo group (Supplementary Table S1).

**TABLE 2:** Primary and secondary outcomes.

Variable	Granisetron (n = 76)	Placebo (n = 74)	Odd ratio or difference (95%CI) d	*p*-Value
28-day mortality, n (%)	26 (34.7), n = 75	26 (35.6), n = 73	0.96 (0.49, 1.89)	0.90
ICU LOS (d, median, IQR)	6 (4,11)	7 (4.25,11)	0 (-2, 1)	0.62
Duration of vasopressors, (h, median, IQR)^a^	44.0 (29.0,99.5), n = 43	37.5 (22.3,56.8), *n* = 46	6 (-8, 23)	0.38
Duration of CRRT (h, median, IQR)^b^	76.5 (38.8,127.5), n = 36	112 (56,146), *n* = 41	-20 (-47.0, 11.0)	0.30
Duration of mechanical ventilation (h, median, IQR)^c^	121.0 (72,184), n = 61	113.5 (56,170.5), n = 64	13 (-20.0, 44.0)	0.45
Incidence of new organ dysfunction (n, %)	13 (17.1)	11 (14.9)	1.18 (0.49, 2.84)	0.88
Change of SOFA in 96 h (mean, SD)	From 9.3 ± 4.2 to 7.4 ± 4.1	From 9.7 ± 4.4 to 8.3 ± 4.8	0.19 (-0.6, 0.97)	0.67
Procalcitonin reduction rate in 96 h (%, median, IQR)	78.6 (53.8,90.3)	72.7 (60.0,86.5)	4.1 (-2.2, 10.4)	0.18

^a^ Excluding patients without vasopressor support in whole ICU stay.

^b^ Excluding patients without CRRT, in whole ICU stay; CRRT, continuous renal replaced treatment.

^c^ Excluding patients without mechanical ventilation in whole ICU stay. The change of SOFA score in 96 h is mainly calculated using a mixed linear model. The mixed model included the baseline SOFA score, time (24, 48, 72, and 96 h), group (granisetron or placebo), and the interaction between group and time, testing the mean difference of SOFA score between groups were the same over time.

^d^ Odd ratios were reported for categorical variables and differences between groups were reported for continuous variables.

Figure 2B shows the outcomes of the subgroup analyses. A significant interaction between the infection site and trial intervention was detected (*p* = 0.02). Specifically, granisetron treatment was associated with an increased risk of mortality compared with the risk in the placebo group for patients with abdominal or digestive tract infections (OR 6, 95% CI 1.10–47.84). Meanwhile, granisetron tended to increase the duration of MV (median difference: 47.5h; 95%CI 0–103) and vasopressors (median difference: 28.3h; 95%CI 4.0–70.0) in this subgroup. For patients with non-abdominal or non-digestive tract infections, the absolute number of 28-day mortality was 10.9% lower in the granisetron group than in the control group (OR 0.63, 95% CI 0.29–1.34). No significant differences in 28-day mortalities were detected in the subgroup analyses stratified by APACHE II score more than 25 or not, age older than 65 years old or not and time of sepsis diagnosis more than 48 h or not.

The attending physician and researcher recorded 95 possible adverse events during the study, but no serious adverse events were reported (Table 3). The adverse events included no defecation for several consecutive days in 69 patients (33 vs. 36), elevated transaminases in six patients (2 vs. 4), and stomach retention in two patients (1 vs. 1). Additionally, one case of new-onset ventricular tachycardia was reported in the control group and one case of paralytic intestinal obstruction was reported in the granisetron group. No significant differences in adverse events were detected between the two groups. However, in the granisetron treatment group, three patients dropped out of the trial due to abdominal distension, and one patient dropped out because of paralytic intestinal obstruction. Continued use of granisetron was deemed inappropriate in the four patients by their attending physicians.

**TABLE 3:** Adverse events.

Adverse events	Granisetron (n = 77)	Placebo (*n* = 77)	*p*-Value
Abdominal distention	6	7	0.77
No defecation for several consecutive days^a^	33	36	0.63
Diarrhea	1	2	0.56
Elevated transaminase	2	4	0.40
Ventricular tachycardia	0	1	0.32
Gastric retention	1	1	1
Paralytic ileus	1	0	0.32

^a^ No defecation for consecutive days means no defecation for at least two consecutive days or more. All patients were included in the safety analysis. The adverse events refer to any adverse effect or unexpected symptoms newly emerging during the trial and probably related to the study intervention (granisetron or placebo). The adverse events were found and reported by the investigator or treatment physicians. Three patients in the granisetron group dropped out of the trial due to abdominal distension and one patient in the granisetron group dropped out due to paralytic intestinal obstruction and was assessed as inappropriate to continue using granisetron.

## Discussion

In the adjuvant treatment of sepsis, there is few successful anti-inflammatory interventions by so far. Nevertheless, inhibiting the excessive inflammatory response is still regarded as an important potential approach and continue to be explored. Evidence from prospective clinical trials demonstrates that 5-HT3 receptor antagonists have an anti-inflammatory effect in inflammatory diseases (Fakhfouri et al., 2012). In a double-blinded trial, the 5-HT3 receptor antagonist, tropisetron, exhibited an equal effect to methylprednisolone in patients with arthritis and activated osteoarthritis (Samborski et al., 2004). In another small trial, granisetron displayed an immediate, short-lasting, alleviating effect in patients with temporomandibular joint inflammatory arthritis (Voog et al., 2004; Fakhfouri et al., 2012). However, only evidence from retrospective study supports the use of 5-HT3 receptor antagonists for the treatment of sepsis. In a retrospective cohort with a large sample size, ondansetron use was associated with reduced in-hospital mortality in patients with acute kidney injury (AKI) and sepsis (Tao et al., 2021). Unlike interleukin-1 receptor antagonist or monoclonal antibody to human tumor necrosis factor alpha biologics in treatment of sepsis, granisetron exerts its effect by suppressing the inflammatory signaling pathway, rather than acting on just one downstream mediator of inflammation, which allows it to play a more important role in the immunoregulation of sepsis. To our knowledge, the GRANTISS trial is the first randomized controlled clinical trial to explore the efficacy of 5-HT3 receptor antagonists in patients with sepsis. The results of the trial showed that granisetron did not reduce the 28-day all-cause mortality of patients with sepsis nor did granisetron improve secondary outcomes compared with placebo.

The study was conducted in a large teaching tertiary hospital, most of the patients were referred from secondary hospitals due to their critical illness, which may have contributed to a higher mortality than reported in other studies. A high proportion of patients completed the planned trial intervention in the study; only a few patients were lost to follow-up. In this trial, a maximum safe dose (3 mg every 8 h) in the instruction was administrated in the granisetron group. The single dose of granisetron in most of clinical trials was 3 mg (Tsuji et al., 2012; Saito et al., 2013; Kitayama et al., 2015). For the effects of granisetron in inflammatory diseases, 3 mg of granisetron was also adopted in the clinical trial on temporomandibular joint arthritis (Voog et al., 2004). In addition, in the retrospective cohort of ondansetron in critical ill patients with AKI, ondansetron use was associated with reduced in-hospital mortality in patients with AKI and sepsis (Tao et al., 2021). Although the specific dose was unclear in this retrospective study, the indications of ondansetron is antiemetic, it is rare to take more than the recommended dose in clinical practices. Another retrospective cohort study also showed that a regular dose of ondansetron rather than a dose exceeding the manufacturers currently recommendation has a positive effect on in-hospital mortality in critical ill patients (Fang et al., 2022). Granisetron and ondansetron are both 5-HT3 antagonists which have the same pharmacological effects. Thus, the dosage regimen in our study should be considered appropriate and reasonable.

According to the results, granisetron did not improve the mortality of patients with sepsis. Additionally, significant effects of granisetron were not detected in three prespecified subgroups analyses. The acute phase of sepsis is characterized by a “genomic storm,” which activates a large number of genes encoding inflammatory cytokines, signal transducers, and cell adhesion molecules, followed by a surge in inflammatory cytokines. These effects culminate in multiple organ dysfunction and early death (DiGiandomenico et al., 2014; Chousterman et al., 2017; Hawkins et al., 2018). Early resolution of the systemic inflammatory response syndrome could rapidly reverse organ dysfunction and restore immune homeostasis to reduce the risk of early death (Hawkins et al., 2018). Although patients received trial intervention as early as possible, some patients may have passed the acute phases when admitted into the ICU, which may impact the anti-inflammatory effects of granisetron. Considering the inhibition of gastrointestinal motility by granisetron, we added a *post hoc* subgroup analysis based on whether the septic patients had abdominal/digestive tract infections, because we observed that septic patients with abdominal/digestive tract infections had a higher probability of gastrointestinal injury. The results showed that there is a significant interaction between the infection site and the trial intervention (*p* = 0.02). For patients without abdominal/digestive tract infections, granisetron intervention tended to improve mortality. Although the statistical power to test the 10.9% difference in mortality was insufficient with this sample size (n = 116), this result still indicates that patients without abdominal or digestive tract infections, rather than all patients, should be considered as a better selection in future studies. Thus, the efficacy of granisetron in this population is still worthy of further determination.

In the analysis of secondary outcomes, granisetron did not improve organ function, PCT reduction rate, or laboratory variables. In our previous studies, a significant negative correlation was shown between granisetron levels and liver injury markers in septic patients (Gong et al., 2019). Additionally, a recent animal study showed that granisetron can reduce sepsis-related liver damage by inhibiting excess inflammation, oxidative stress, and cell pyroptosis. These results suggest that the greatest protective effects of granisetron are in the liver (Aboyoussef et al., 2021). However, no statistical differences in liver injury biomarkers were detected between granisetron and placebo in this study. Endogenously generated granisetron levels were not measured in this study. Hence, the impact of endogenously produced granisetron on exogenous granisetron treatment is unclear. The correlation between the level of endogenous granisetron and the degree of organ dysfunction in septic patients should be explored in future studies to determine whether granisetron treatment in sepsis is beneficial and to determine the optimal septic patients for treatment.

The occurrences of adverse events were not significantly different between the two groups, suggesting that granisetron is generally safe to use in patients with sepsis. However, in the subgroup analysis of patients with abdominal/digestive tract infections, granisetron negatively affected prognosis. Although the small sample (n = 32) may cause a contingency, this effect also may be related to the inhibition of gastrointestinal motility by 5-HT3 receptor antagonists (PRIOR & READ, 1993; Gershon, 2004). Abdominal/digestive tract infection is major risk of gastrointestinal dysfunction, the inhibition of gastrointestinal motility by 5-HT3 receptor antagonists could further affect the gastrointestinal function (Yang et al., 2013; Frazer et al., 2018). In future studies, granisetron should be used cautiously in patients with abdominal/digestive tract infections.

In the GRANTISS study, we initially explored the effectiveness and safety of granisetron in patients with sepsis. To the best of our knowledge, this is the first evidence from a prospective clinical trial concerning the effects of 5-HT3 receptor antagonists on sepsis. As an explorative trial, in order to detect and deal with possible adverse reactions of granisetron in time, we selected a single-blinded study over a double-blinded study. Nevertheless, we still implemented the blinding by using the placebo with the same appearance as granisetron and same injection container with the same label. Moreover, the study investigators were not involved in the routine treatment decisions, which minimized the bias caused by a single-blind study. However, the results still should be properly interpreted due to study limitations. First, this was an exploratory single-center study hence, it was underpower to detected a small difference in mortality at current sample size. Second, the patients who dropped out or were lost to follow-up may lead to a slight attrition bias. Third we only evaluated the SOFA score and laboratory variables for 96 h, and the long-term effects of granisetron on the above variables remain unclear.

## Conclusion

In summary, in the GRANTISS trial, granisetron did not improve 28-day mortality and the related prognosis of patients with sepsis. However, as the numerically lower mortality in septic patients without abdominal/digestive tract infection in granisetron group, further clinical trials with larger sample size are worthy of consideration to determine the efficacy of granisetron in patients with sepsis without abdominal/digestive tract infections.

## Data Availability

The original contributions presented in the study are included in the article/Supplementary Material further inquiries can be directed to the corresponding authors.
