# Supplementary material for: Adjunctive granisetron therapy in patients with sepsis or septic shock (GRANTISS): A single-center, single-blinded, randomized, controlled clinical trial
Source: Front Pharmacol. 2022 Dec 13;13:1013284. doi: 10.3389/fphar.2022.1013284 (PMC9792607; doi:10.3389/fphar.2022.1013284)
Supplement: Supplementary file 1 [file DataSheet1.docx]

**Supplemental material**

**Adjunctive granisetron therapy in patients with sepsis or septic shock (GRANTISS): a single-center, single-blinded, randomized, controlled clinical trial**

**Content**

1. **Study protocol for GRANTISS trial………………………………………….2**
2. **Statement of amendments to the protocol……………………………………2**
3. **Sepsis 3.0 diagnostic criteria…………………………………………………..2**
4. **Supplement Table1. The mean differences in laboratory indicators between groups……4**
5. **Supplement Figure 1. Kaplan-Meier survival curve during the 60-day follow-up……….5**
6. **Supplement Figure 2. The box plot of Mean Sequential Organ Failure Assessment (SOFA) Scores over time……………………..…………………..5**
7. **Study protocol for GRANTISS trial**

The study protocol was previously published. See the article entitled, “Adjunctive granisetron therapy in patients with sepsis or septic shock (GRANTISS): Study protocol for a randomized controlled trial” for more details. (doi: 10.1097/MD.0000000000017354)

1. **Statement of amendments to the protocol**
2. **Statement of amendment to dilution protocol**

According to the instructions for granisetron, intravenous infusion is recommended after dilution with 20–50 ml of normal saline. Thus, we adjusted the dilution volume. The specific protocol was adjusted as follows:

**Previous trial intervention:** Patients in the granisetron group will receive granisetron 3 mg (PKU HealthCare Corp, Ltd) diluted to **15 mL** with **12 mL** of saline and intravenously administered within 10 min. Patients in the placebo group will receive **15 mL** of normal saline administered intravenously within 10 min.

**Revised trial intervention:** Patients in the granisetron group will receive granisetron 3 mg (PKU HealthCare Corp, Ltd) diluted to **25 mL** with **22 mL** of saline and intravenously administered within 10 min. Patients in the placebo group will receive **25 mL** of normal saline administered intravenously within 10 min.

**Execution date of the previous plan:** April 25, 2019, to April 27, 2019

**Execution date of the adjusted plan:** April 27, 2019, to trial termination

**(2) Statement of the amendments to subgroup analyses**

Considering the inhibition of gastrointestinal motility by granisetron, we revised the subgroup analysis to determine whether abdominal/digestive tract infections affected the outcomes. Additionally, during the trial, when we evaluated the baseline SOFA scores, we found that most patients enrolled in the group had more than two organs damaged. The increase in sofa score comes from at least two organ injuries. Thus, we canceled the subgroup analysis based on multiple organ dysfunction.

**Execution date of the previous plan:** April 25, 2019, to November 1, 2020

**Execution date of the adjusted plan:** November 1, 2020, to trial termination

**3. Sepsis 3.0 diagnostic criteria**

In sepsis 3.0 diagnostic criteria, sepsis was defined as life-threatening organ dysfunction caused by a dysregulated host response to infection. Specifically, the diagnostic criteria are as the following: 1) with suspected infection; 2) with an acute increase in the Sequential Organ Failure Assessment (SOFA, see table1 for details) of 2 points or more consequent to infection, and the baseline SOFA score can be assumed to be zero in patients not known to have preexisting organ dysfunction [1]. For clinical operationalization, serum procalcitonin (PCT) was used as an important marker to judge suspected infection in this study, especially PCT≥2ng/ml can further improve the specificity of judgment [2,3].

**Table. Sequential Organ Failure Assessment Score.**

| **System** | **Score** | | | | |
| --- | --- | --- | --- | --- | --- |
|  | **0** | **1** | **2** | **3** | **4** |
| **Respiration**  PaO2/FiO2,mmHg (Kpa) | >=400 | <400 | <300 | <200 with respiratory support | <100 with respiratory support |
| **Coagulation**  Platelets, *10^3/μl | >=150 | <150 | <100 | <50 | <20 |
| **Liver**  Bilirubin, mg/dL | <1.2 | 1.2-1.9 | 2.0-5.9 | 6.0-11.9 | >12.0 |
| **Cardiovascular** | MAP>=70mmHg | MAP<70mmHg | Dopamine <5 or dobutamine (any dose) | Dopamine 5.1-15  or epinephrine <=0.1 or norepinephrine <=0.1 | Dopamine >15 or epinephrine >0.1 or norepinephrine>0.1 |
| **Central nervous system**  Glasgow Coma Scale score | 15 | 13-14 | 10-12 | 6-9 | <6 |
| Renal  Creatinine, mg/dL(μmol/L)  Urine output, ml/d | <1.2 | 1.2-1.9 | 2.0-3.4 | 3.5-4.9  <500 | >5.0  <200 |

Reference

1. Singer M, Deutschman CS, Seymour CW, et al. The Third International Consensus Definitions for Sepsis and Septic Shock (Sepsis-3). JAMA. 2016;315(8):801-810.
2. Marik PE, Khangoora V, Rivera R, Hooper MH, Catravas J. Hydrocortisone, vitamin C, and thiamine for the treatment of severe sepsis and septic shock: a retrospective before-after study. Chest 2017;151:1229-38.
3. Pontrelli G, De Crescenzo F, Buzzetti R, et al. Accuracy of serum procalcitonin for the diagnosis of sepsis in neonates and children with systemic inflammatory syndrome: a meta-analysis. BMC Infect Dis. 2017;17(1):302. Published 2017 Apr 24.

**4. Supplement Table 1. Mean differences in laboratory indicators between groups over 96 h**

| **Laboratory indicators** | **Mean differences between groups**  **after adjusted baseline (MD, 95% CI)** |  |
| --- | --- | --- |
| alanine transaminase, IU/L | 69.2 (−65.1, 203.6) |  |
| aspartate aminotransferase, IU/L | 124.4 (−436.4, 685.1) |  |
| Total bilirubin, umol/L | −1.4 (−17.6, 14.8) |  |
| Interleukin-6, ng/L | 194.0 (−90.5, 478.4) |  |
| C-reactive protein, ng/L | 5.1 (−15.7, 25.8) |  |
| Erythrocyte sedimentation rate, mm/h | −0.72 (−12.2, 10.8) |  |
| White blood cell, 10^9/L | 1.4 (−0.8, 3.5) |  |
| lymphocyte, 10^9/L | −0.12 (−0.6, 0.3) |  |
| Cystatin C, mg/L | 0.13 (−0.14, 0.41) |  |
| Serum creatine, μmol/L | 15.53 (−7.73,38.79) |  |
| Blood urea nitrogen, mmol/L | −2.23 (−6.94,2.48) |  |
| Blood lactate, mmol/L | −0.6 (−1.2, 0.1) |  |
| Procalcitonin, ng/ml | -2.8 (-11.2, 5.6) |  |
| Superoxide dismutase, ku/L | −6.3 (−15.8, 3.3) |  |
| PaO2/FIO2 | −1.71 (−26.9, 23.5) |  |
| CD4^+^ T cell count ^a^, /μL | -2.4 (-105.0, 100.4) |  |
| CD8^+^ T cell count ^a^, /μL | 17.3 (-48.1, 82.7) |  |
| Fungi (1,3)-beta-D-Glucan ^a^,μg/L | -0.10 (-0.50, 0.30) |  |
| Galactomannan antigen ^a^ | -16.6 (-41.1, 7.8) |  |

The mean difference of each laboratory indicator during the study intervention period was calculated using the mixed linear model. The mix model included the baseline level, the time points (24, 48, 72, and 96 h), groups (granisetron or placebo), and the interaction between groups and time. MD > 0 indicates that the mean level of the indicator in the granisetron group is higher than that in the placebo group. When p < 0.05, the difference between the groups is statistically significant. MD = mean difference; 95% CI = 95% confidence interval.

^a^ These indicators were only tested on the baseline and at 96h, therefore, the corresponding model included the baseline level, group (granisetron or placebo).


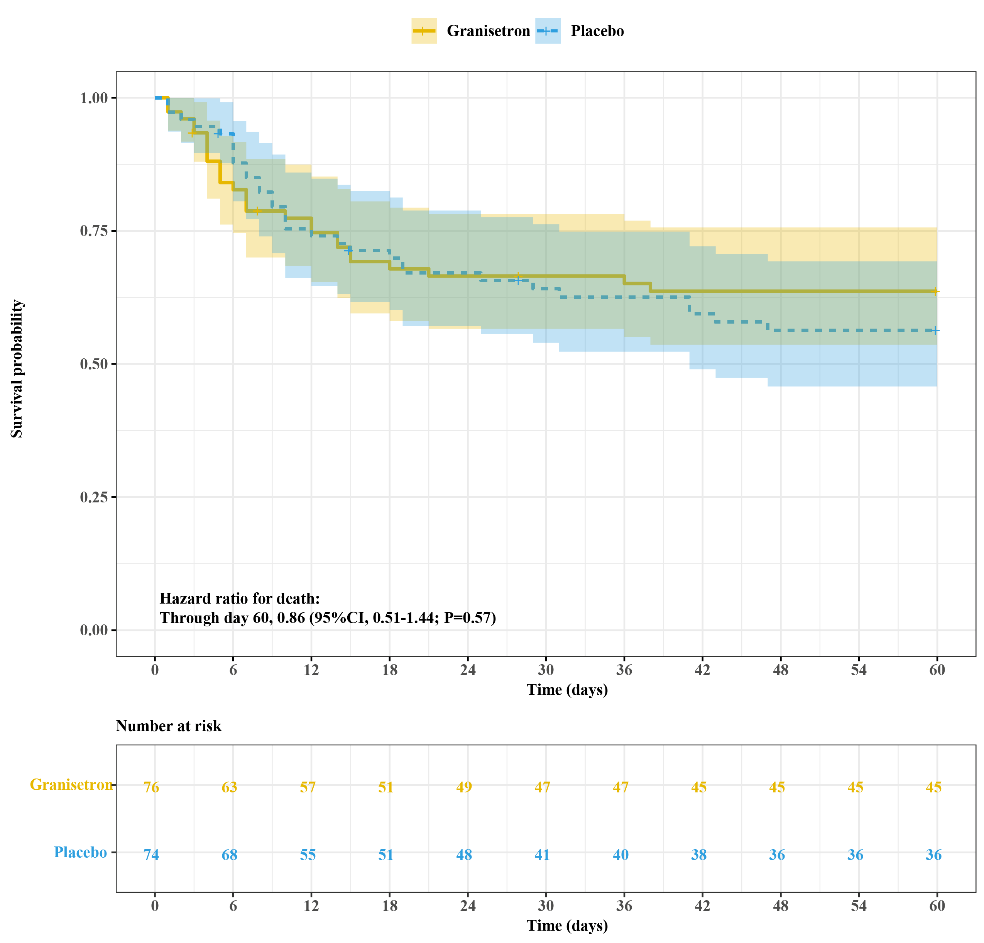


**5.Supplement Fig. 1. Kaplan-Meier survival curve during the 60-day follow-up.** The tick marks indicated censored data. The P value was calculated using a Cox proportional hazards model that included the randomized trial group.

**
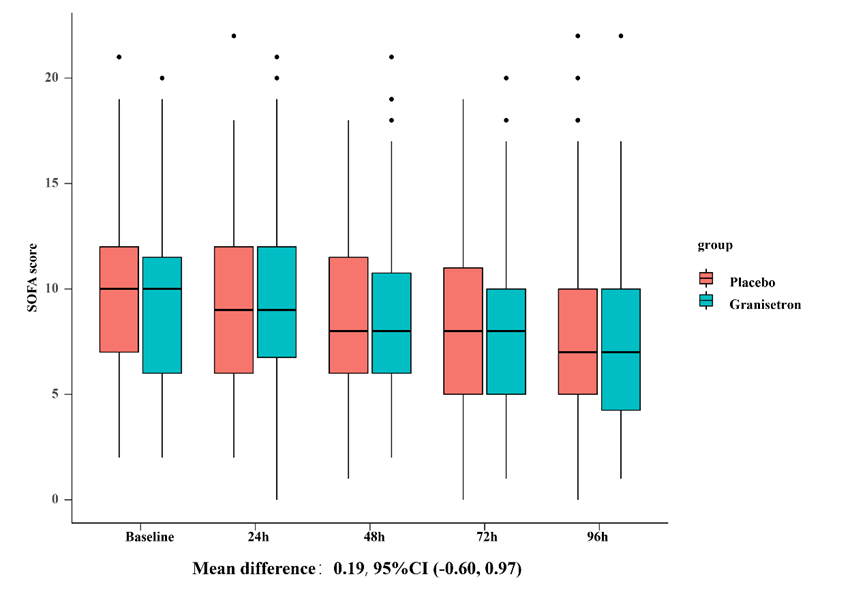
**

**6. Supplement Fig. 2. Box plot of mean SOFA (Sequential Organ Failure Assessment) scores over time.** SOFA scores of patients during the study intervention are shown. The horizontal line inside the box represented the median values, the top and bottom of boxes represent the interquartile range, and the points beyond the top of the vertical lines are outliers.
